# Cereal–Legume Food Matrices as Functional Systems: Processing-Driven Synergies in Nutrition, Bioactive Compounds and Sensory Acceptability

**DOI:** 10.3390/molecules31122033

**Published:** 2026-06-10

**Authors:** Shonisani Eugenia Ramashia, Mmaphuti Abashone Ratau, Gbeminiyi Olamiti

**Affiliations:** Department of Food Science and Technology, Faculty of Science, Engineering and Agriculture, University of Venda, Thohoyandou 0950, South Africa; shonisani.ramashia@univen.ac.za (S.E.R.); mmaphuti.ratau@univen.ac.za (M.A.R.)

**Keywords:** cereal–legume systems, food matrix interactions, processing technologies, bioactive compounds, nutrient bioavailability, sensory acceptability

## Abstract

As global trends continue to embrace environmentally friendly, plant-based diets, food systems that are nutrient-dense, climate-resilient, and economically viable in addressing protein–energy malnutrition, micronutrient deficiencies, and food insecurity have increased. Although cereal–legume combinations are widely recognised to be highly nutritious, most studies have focused primarily on enhancing compositional efficiency and have overlooked their interactions with the food matrix and the processing-mediated transformations they undergo. This review combines recent findings examining cereal–legume food matrices as functional systems, with particular emphasis on nutritional complementarity, bioactive interactions, processing-induced modifications, and sensory acceptability. Studies indicate that cereals and legumes provide complementary amino acid profiles, dietary fibre, essential micronutrients, and phytochemicals within these composite matrices that influence digestibility, bioavailability, antioxidant activity, and glycaemic response. Processing methods, including fermentation, germination, roasting, and extrusion, modulate these interactions by releasing bound phenolics, reducing antinutritional factors, and altering starch–protein–phenolic complexes, thereby affecting health functionality and sensory quality. However, inadequately optimised processing can affect nutrient retention and consumer acceptability. Overall, this review emphasises the relevance of integrating food matrix science and processing optimisation for the production of functional, acceptable, and sustainable cereal–legume foods that promote product innovation, public health improvement, and the utilisation of underutilised crops for sustainable food systems.

## 1. Introduction

The global food system is in the midst of an unprecedented transformation, driven by a greater understanding of the long-term environmental, nutritional, and health consequences. There is a growing trend towards plant-based diets in response to threats such as non-communicable diseases (NCDs), including obesity, diabetes, and cardiovascular disorders, as well as global warming [1,2]. From a plant-based dietary perspective, nutrient-dense foods are promoted, such as cereals, legumes, fruits, and vegetables, which are rich in dietary fibre, bioactive compounds, and essential micronutrients. In this state, cereal–legume combinations are emerging as affordable, readily available food systems that complement the nutrient requirements of diets to combat protein–energy malnutrition and micronutrient deficiencies in low-and middle-income countries (LMICs). Indigenous cereals such as sorghum, millet, teff, and fonio, and legumes such as cowpea, Bambara groundnut, pigeon pea, and chickpea are essential components of traditional diets in Africa and Asia. They are naturally adaptable to harsh weather such as drought, poor soils, and high temperatures, and are essential for climate-smart agriculture and food security [1,2].

In terms of nutrition, cereals contain carbohydrates, dietary fibre, and some vitamins, while legumes are high in proteins, lysine, and bioactive phytochemicals. Their contrasting amino acids are found in cereals, which are low in lysine and high in sulphur-rich amino acids, and, with the exception of legumes, this is the biochemical basis for improving protein quality when cereals and legumes are combined [3]. Aside from macronutrients, they have a wide range of bioactive constituents, including phenolics, flavonoids, and resistant starches, all of which exert effects on antioxidant capabilities, glycaemia, and gut health [4].

However, more traditional studies have mainly centred on single-crop comparisons, comparing cereals and legumes for their proximate composition, functionality, and nutritional effects. While such methods provide a basic understanding, they tend to overlook the fact that, in real food systems, components interact within a matrix. Single-crop assessments neglect key interactions, including protein–starch complexation, phenolic–protein interactions and fibre-mediated encapsulation, which affect bioavailability, digestibility, and functional performance of nutrients [5]. Furthermore, these component-focused strategies lack adequate consideration of the influence of conventional food processing methods, such as fermentation, germination, roasting, and extrusion, on such interactions and, consequently, on the quality of the food products obtained.

Increasing evidence indicates that food matrices are fundamental to understanding the nutritional and functional characteristics of composite foods. Food matrices are the physical and chemical properties of the elements in a food system that influence nutrient release, bioaccessibility, and physiological responses [6]. In cereal–legume systems, such interactions may appear synergic, and the effect can be greater than the sum of the components. For example, fermentation can also enhance phenolic bioavailability via enzymatic hydrolysis whilst decreasing antinutritional factors such as phytates and tannins, thereby promoting mineral absorption [7]. Germination, on the other hand, activates endogenous enzymes that modify starch and protein structures, thereby improving digestibility and functional properties. Extrusion processing has also been shown to have additional beneficial effects on structuring macromolecules, promoting starch gelatinisation and protein denaturation, and enhancing phenolic accessibility and the glycaemic response once optimised [8].

An underlying justification for adopting a synergy-based approach is the need to go beyond the optimisation of composites to the system-level understanding of food functionality. Synergism-based frameworks focus not just on adding nutrients and fortifying, but on optimising interactions to enrich the balance and sensory benefits of these nutrients through processing. This understanding also aligns with a trend in the current functional food development literature, which focuses on bioavailability, health-enhancing characteristics, and consumer acceptability. Importantly, this approach is especially salient for underutilised crops, the full potential of which remains underutilised because they are not integrated into modern food systems and cannot be understood or used due to their interactive behaviour in composite formulations.

Sensory acceptance is an important factor influencing consumers’ acceptance of cereal–legume products. It is imperative for nutritional improvement, as texture, flavour, colour, and overall sweetness are critical determinants of marketing effectiveness. Processing methods have a dual nature, as they can enhance or diminish certain sensory experiences under certain circumstances. For example, controlled fermentation can enhance the complexity of flavours and remove undesirable sensory attributes in legumes; however, excessive heat treatment may lead to deterioration of colour and nutrients. Therefore, creating a sustainable and appealing food product necessitates a thorough nutritional, functional, and sensory evaluation. In this context, the present work reviews existing knowledge regarding cereal–legume food matrices as functional systems, emphasising the synergies driven by processing in terms of nutrition, bioactive compounds, and sensory acceptance. This study explored how cereals and legumes complement each other nutritionally and in terms of bioactive properties within composite matrices. It assessed the effects of various processing methods, such as fermentation, germination, roasting, and extrusion, on interactions within these matrices and nutrient bioavailability. Additionally, it investigated how interactions among bioactive compounds influence health benefits while also considering their impact on sensory quality and consumer preference. This study advances recent developments in the field of food matrices, particularly regarding underutilised crops from sub-Saharan Africa and other developing regions that hold potential to foster a sustainable food system, enhance nutritional value, and promote economic growth.

This review specifically focuses on cereal–legume food matrices relevant to sub-Saharan Africa, with emphasis on commonly consumed and underutilised cereals such as sorghum, pearl millet, finger millet, maize and indigenous maize varieties, and legumes such as cowpea, Bambara groundnut, soybean, pigeon pea, common bean and African yam bean. The review prioritises composite systems used in porridges, complementary foods, composite flours, fermented products and extruded snacks because these products are nutritionally important, culturally acceptable and technologically feasible in African food systems. The geographical focus on sub-Saharan Africa is justified by its high reliance on cereal-based diets, persistent protein and micronutrient deficiencies, postharvest losses, and the growing need for affordable value-added foods. Processing methods considered in the review include soaking, dehulling, germination, fermentation, roasting, cooking, parboiling, extrusion, and selected emerging technologies where these methods influence nutrient bioavailability, bioactive compound release, antinutrient reduction, food matrix structure, sensory quality, and consumer acceptability.

## 2. Methodology

The authors employed a narrative review methodology, supported by a structured, transparent literature search strategy, to compile and evaluate evidence on cereal–legume food matrices as functional systems. The review focused on processing-driven synergies related to nutrition, bioactive compounds, nutrient bioavailability, food matrix interactions, functional properties, and sensory acceptability. Although the study was not designed as a systematic review or meta-analysis, the search, screening, and synthesis procedures were organised to improve transparency, reproducibility, and interpretive rigour (Table 1).

The literature search strategy was conducted in Scopus, Web of Science, PubMed, ScienceDirect, and Google Scholar for publications from 2000 to 2026, with earlier foundational papers included only when they provided essential conceptual or mechanistic information. The following combined terms were searched: “cereal–legume composites”, “food matrix interactions”, “processing effects”, “bioactive compounds”, “phenolic interactions”, “protein digestibility”, “mineral bioavailability”, “functional foods”, “fermentation”, “germination”, “extrusion”, “sensory acceptability”, “consumer acceptance” and “sub-Saharan Africa”. Boolean operators such as AND and OR were used. Reference lists of selected articles were also manually screened to identify additional relevant studies.

The screening process, including titles and abstracts, was first screened for relevance to cereal–legume composite systems, processing technologies, nutrition, bioactive compounds, functional properties, and sensory or consumer-related outcomes. Full texts of the articles were then assessed to confirm whether the studies addressed composite food matrices rather than isolated raw materials. Studies were retained when they provided evidence on at least one of the following outcomes: nutrient composition, protein quality, amino acid complementarity, mineral bioavailability, antinutrient reduction, phenolic or flavonoid modification, antioxidant activity, starch or protein digestibility, rheological or functional behaviour, sensory quality, consumer acceptability, or product development potential.

Data extraction and synthesis were classified thematically according to cereal–legume combination, geographical context, processing method, product type, nutritional outcome, bioactive compound response, antinutritional factor reduction, functional property, sensory attribute, and consumer acceptability indicator. The extracted evidence was synthesised qualitatively by comparing patterns across studies and identifying recurring processing-driven mechanisms, including protein–starch–phenolic interactions, enzymatic hydrolysis during fermentation and germination, structural modification during extrusion, and heat-induced changes during roasting or cooking. Because of heterogeneity in crop types, formulations, processing conditions, and analytical methods, a quantitative meta-analysis was not conducted.

The scope and justification of the study were deliberately framed around sub-Saharan African cereal–legume systems because these foods are central to household diets, complementary feeding, small-scale processing, and emerging value-added food enterprises in the region. The review, therefore, prioritises cereal–legume combinations involving cowpea, Bambara groundnut, soybean, pigeon pea, common bean, and African yam bean. This scope allows the study to connect nutritional improvement with practical processing methods, cultural relevance, sensory acceptability, and scalability within African food systems.

## 3. Indigenous Cereals and Legumes: An Overview

### 3.1. Indigenous Cereals and Legumes

Indigenous cereals are traditional grains that have been grown for generations and play an important role in the diets of people in Africa, Asia, and certain regions of Latin America [9]. Traditional grains such as sorghum (*Sorghum bicolor*), millet (*Eleusine coracana*), pearl millet (*Pennisetum glaucum*), maize (*Zea mays*), and various traditional rice (*Oryza sativa*) varieties are also recognised as indigenous cereal types [10]. Indigenous cereals, which thrive in areas characterised by severe dryness, bad soil quality, and dense populations, are particularly resilient. Smallholder farmers often grow these crops on limited land while intercropping with other plants. These cereals usually align well with conventional agricultural practices and adapt to local soil, rainfall, and wind conditions. In addition to their resilience, indigenous cereals are rich sources of essential nutrients, making them an important part of the diet; they provide most of the carbohydrate energy and also supply significant amounts of protein, dietary fibre, and micronutrients such as zinc, iron, and B vitamins. Some indigenous cereals, such as millet and sorghum, also contain high levels of bioactive phytochemicals, including flavonoids and antioxidants, which are linked to positive health effects, including improved digestibility, better prevention of chronic diseases, and enhanced metabolic health [10,11].

In addition to the nutritive value, indigenous cereals may possess various functional properties that can be applied in processing and formulation. Such properties are related to the various starch fractions in the grains and are related to water absorption, gelatinisation, gel formation, viscosity, and other functional properties that are relevant to the formulation and processing of various indigenous traditional foods and beverages, such as porridges, bread, biscuits, and fermented beverages [1]. Indigenous cereals can be subjected to malting, fermentation, and germination, thereby improving their nutritive value, textural properties, and digestibility [12,13].

Besides their nutritive benefits, cereal proteins have the disadvantage of being deficient in several essential amino acids, such as lysine. Therefore, the relationship between cereals and other foods (mainly legumes) should be exploited to produce nutritionally adequate products. In fact, a complementary amino acid profile is achieved by mixing cereals with other food ingredients, particularly legumes high in lysine, thereby enhancing the nutritional value of the diet. This practice has been well known in traditional diets for many centuries; nowadays, this principle is being employed in the development of new food products associated with health benefits, known as functional foods [13,14].

Indigenous legumes are long-standing pulse crop varieties widely cultivated in Africa, Asia, and Latin America. These include crops such as cowpea (*Vigna unguiculata*), Bambara groundnut (*Vigna subterranea*), pigeon pea (*Cajanus cajan*), soybean (*Glycine max*), and African yam bean (*Sphenostylis stenocarpa*). Indigenous legumes are resilient across various agroecological regions and are major crops in semi-arid and drought-prone environments [15,16]. Many indigenous legumes are hardy crops cultivated by smallholder and rural farmers and adapt well to low-fertility soils. Traditionally, they serve as vital sources of nutrition, as they are high in plant protein, complex carbohydrates, and dietary fibre; they are central to a healthy diet and rich in essential micronutrients such as iron, calcium, magnesium, and zinc, as well as B-vitamins like folate. Some indigenous varieties can also surpass many cereals in protein content. These leguminous crops offer high fibre content, supporting digestion and potentially helping to prevent non-communicable diseases such as diabetes and cardiovascular diseases [17,18,19].

The local use of pulses in cooking is significant, as these ingredients play a crucial role in food processing and product innovation. They possess functional characteristics, including water retention, emulsification, gel formation, and oil binding, which affect the texture and stability of foods over time. Indigenous pulses are used in composite flour blends for the processing of porridges and bakery products, as snack foods, and in the fermentation of foods. Traditional processing techniques such as soaking, fermentation, and germination are used in local dishes to improve digestibility, minimise antinutritional factors, and enhance the bioavailability of minerals and proteins. The biophysical, nutritional, and functional features of indigenous cereals and pulses are varied, and they are always considered complementary sources [20].

Other indigenous cereals such as sorghum, pearl millet, finger millet, indigenous maize flour, and traditional varieties are mainly used as sources of food energy and digestible carbohydrates. These are mostly cultivated in larger quantities, often as an essential part of the staple diet and as important sources of calories [21]. However, a cereal contribution of only 20% may be insufficient to achieve meaningful nutritional complementarity, particularly because most cereal proteins are deficient in the essential amino acid lysine. Other indigenous feeds and legumes, such as cowpea, soybean, indigenous varieties of Bambara groundnut, and pigeon pea, provide a superior plant protein source and contain higher amounts of dietary fibre and trace elements, which are generally marginally higher in protein and less affected by antinutrients such as phytates, tannins, and trypsin inhibitors [15,16,17].

### 3.2. Comparative Nutritional Attributes

Indigenous cereals and legumes are complementary food resources central to traditional and contemporary diets in sub-Saharan Africa and other developing regions. Cereals such as sorghum (*Sorghum bicolor*), pearl millet (*Pennisetum glaucum*), finger millet (*Eleusine coracana*), maize (*Zea mays*) and traditional rice varieties provide dietary energy, starch, fibre, B-vitamins and minerals, while legumes such as cowpea (*Vigna unguiculata*), Bambara groundnut (*Vigna subterranea*), soybean (*Glycine max*), pigeon pea (*Cajanus cajan*), common bean (*Phaseolus vulgaris*) and African yam bean (*Sphenostylis stenocarpa*) provide protein, lysine, dietary fibre, minerals and bioactive compounds. Their combined relevance is strengthened by their adaptability to smallholder farming systems, semi-arid environments, and low-input agricultural conditions, making them important for food security, dietary diversification, and value-added product development [10,16].

### 3.3. Comparative Functional and Technological Attributes

The nutritional value of these systems is also influenced by antinutritional factors such as phytates, tannins, and trypsin inhibitors, which may reduce mineral bioavailability and protein digestibility [22]. However, processing methods such as soaking, fermentation, germination, cooking, and extrusion can reduce these constraints while improving nutrient release and digestibility [23]. Therefore, the nutritional advantage of cereal–legume blends depends not only on formulation ratio, but also on the processing conditions used to transform raw materials into acceptable foods.

Nutritionally, cereals are major sources of digestible carbohydrates and energy, but their protein content is often low in lysine [1]. Legumes, in contrast, contain higher protein levels, often approximately 20–40% depending on species, and are comparatively rich in lysine, making them suitable for improving the amino acid balance of cereal-based diets [24]. When combined, cereal–legume systems can improve protein quality, enhance dietary fibre intake, and provide essential micronutrients such as iron, zinc, magnesium, calcium, and folate. This complementarity is particularly relevant in regions where cereal-based diets dominate and where animal-source foods may be limited by cost or accessibility [17,18].

### 3.4. The Cereal–Legume Synergy Framework

Functional complementarity supports the development of composite flours, fermented foods, complementary foods, baked products, plant-based foods, and ready-to-eat snacks. For example, cereal starch can provide expansion and crispness during extrusion, while legume protein can improve nutritional density and matrix strength. However, excessive legume inclusion may result in a beany flavour, bitterness, a darker colour or reduced expansion, indicating that functional synergy must be optimised alongside sensory acceptability.

Functionally, cereals and legumes play distinct but complementary roles in food formulation. Cereal starches contribute water absorption, gelatinisation, pasting behaviour, gel formation, viscosity and textural body, which are essential in porridges, breads, biscuits, beverages and extruded products. Legume flours and proteins contribute water absorption, emulsification, foaming, oil binding, gelation, and structural reinforcement, thereby improving product stability, texture, and mouthfeel when properly balanced [1,20].

## 4. Nutritional and Functional Synergies of Indigenous Cereals and Legumes

**Figure 1:**
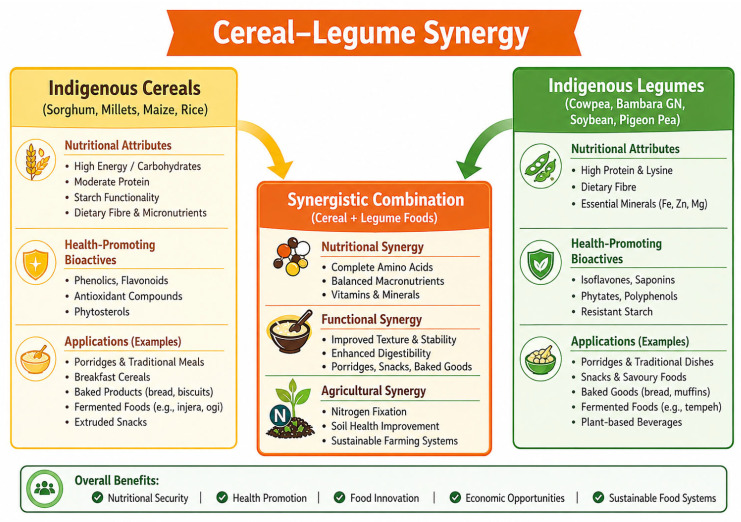
Cereal–legume synergy.

Indigenous cereals such as sorghum, millet, and maize, and many legumes such as cowpeas, bambara groundnuts, and beans have been the cornerstones of traditional diets in many parts of Africa for a very long time. They synergistically enhance the nutritive value, processing functionality, and sustainability of agroecosystems. They are critical for food security, combating malnutrition, and climate change resilience in sub-Saharan Africa, as shown in Figure 1 and Table 2.

### 4.1. Nutritional Complementarity

In regions where plant sources make up a significant portion of daily diets, efforts are being made to improve dietary nutritional content by combining legumes and cereals. Cereal and leguminous foods are rich sources of main energy-providing nutrients such as carbohydrates. Cereals such as sorghum, millets, maize, and rice are good sources of energy, providing carbohydrates, but are deficient in some nutritionally important amino acids, such as lysine. These cereals provide little protein but are rich in sulphur amino acids, such as methionine and cysteine. Legumes such as cowpea, Bambara groundnut, pigeon peas, and soya beans, on the other hand, provide a significant amount of lysine but are limited in sulphur amino acids. Combined cereal and leguminous food products are therefore of high nutritional quality. The physicochemical basis of combined food products is well established, has been demonstrated in traditional foods, and remains highly pertinent to the formulation of modern food products [18,19].

### 4.2. Functional Properties in Food Processing

In addition to their nutritional complementarity, the functional properties of cereals and legumes make them a natural suite for synergistic interactions during food processing and formulation. Cereals can offer a range of desirable starch properties that affect texture, viscosity, and structure, while legume proteins are used for their emulsifying, foaming, and water-binding properties. According to Boye et al. [24], such synergisms enable food technologists to produce a range of synergistic food products, including porridges, snacks, baked goods, and fermented foods, with enhanced texture, functional, and nutritional qualities.

### 4.3. Role in Traditional and Modern Diets

The relationship between cereals and legumes has been a deliberate and recognised aspect of traditional diets in various cultures throughout history. This is evident in many dishes that combine cereal grains such as maize, sorghum, or rice with pulses or nuts like lentils, beans, and groundnuts. Kumari and Sangeetha [33] reported that the widespread intergenerational use of these traditional crop combinations in porridges and rice-based mixtures with legumes reflects deliberate experimentation rather than mere chance. Through their own experiences, nations have identified the combinations that effectively maximise the benefits of these foods while enhancing the enjoyment of meals.

In contemporary times, the integration of cereal–legume pairings into modern diets and cooking methods is advancing through innovative food technology and scientific research, aiming to improve their utility. Today, this combination is increasingly used in the production of composite flour, plant protein foods, fortified products, and ready-to-eat or ready-to-cook foods, fostering nutritional similarity, improving palatability, and increasing acceptability. Culturally, the taste, texture, and sensation of satiety or chewiness experienced when consuming legumes play a crucial role in their popularity. Additionally, considering both environmental and nutritional aspects, the ability of these crops to enhance sustainability further increases the attractiveness of this combination of plant categories [33].

### 4.4. Contribution to Functional and Fortified Foods

There are encouraging initiatives to integrate cereals and pulses (legumes) into functional and fortified foods to address nutrient deficiencies, particularly among vulnerable populations such as infants and children. The combination of cereals and legumes offers synergistic benefits, providing complementary amino acids that enhance protein quality and are rich sources of iron, zinc, and B vitamins. Moreover, legumes provide additional bioactive compounds, including phenolics and dietary fibre, that promote health and aid in disease prevention. These attributes render cereal–legume blends ideal for creating nutritious snacks, instant beverages, baking mixes, fortified porridges, and instant food products abundant in iron, zinc, or protein. Such offerings can act as supplementary foods for infants and young children or be distributed via school and community feeding programmes [15,18].

In addition to nutritional benefits, cereal–legume combinations offer functional advantages that significantly improve food technology acceptance and nutrition. The proteins and soluble components from legumes enhance water absorption and the emulsification and gelation properties of the foods they are incorporated into, leading to improved texture, consistency, and stability. This dimension of food technology is vital for developing acceptable, shelf-stable, and affordable food options for domestic producers in low-income or food-insecure settings. The study by Temba et al. [34] indicates that regular consumption of these foods can markedly boost growth, cognitive development, disease resistance, and overall health among malnourished populations by substantially reducing the incidence of protein–energy malnutrition (PEM) and micronutrient deficiencies. These nutritional advantages position cereal–legume-based functional foods as a sustainable solution to achieving food security across various dietary contexts.

### 4.5. Ecological and Agricultural Synergy

The advantages of combining cereals and legumes extend beyond mere nutritional value; they also play a crucial role in enhancing agricultural sustainability. Legume crops contribute to this by fixing atmospheric nitrogen through their symbiotic partnerships with soil microorganisms. When cereals and legumes are cultivated together or in rotation, the cereals benefit from increased nutrient availability, which results in higher yields without the need for excessive chemical inputs. This approach not only enhances productivity but also reduces environmental risks. In addition to their nutritional benefits, intercropping or rotating these crops offers further advantages, such as improved soil structure, greater biodiversity, and enhanced resilience against pests, diseases, and climate variability. Cereal–legume systems facilitate more effective utilisation of land and resources, promoting sustainable long-term agricultural productivity. Thus, implementing cereal–legume systems is vital for developing environmentally friendly and economically viable production methods that increase global food security by ensuring stable yields, conserving soil health, and fostering resilient farming practices in anticipation of future climate challenges [35].

Overall, the majority of the syntheses suggest strong potential for cereal–legume matrices, but their benefits are also dependent on the extent to which they are processed with controlled efficacy, correctly modulated, and have context-specific sensory and economic validation. Hence, a balanced interpretation should acknowledge the opportunities and constraints of synergy: while it is possible that processing can enhance nutrient release, bioactive accessibility, and product functionality, under poorly optimised conditions, nutrient losses, sensory rejection, and health-related effects can occur [4].

There is also considerable variation in methods in the literature, reducing the potential for direct study comparison. Variations in formulation proportions, analytical procedures, in vitro digestion models, sensory panels, consumer cohorts, processing scales, and reporting formats have inhibited the ability to provide generalised recommendations. Studies are either based on in vitro bioaccessibility assessments or compositional indicators, rather than in vivo validation, and long-term acceptance by consumers, household preparation methods, and economic feasibility are not well studied. Therefore, reports on metabolic health, modulation of the gut microbiota, and disease prevention should be considered suggestive rather than conclusive until confirmed by stronger biological and population-level evidence [36].

Sensory trade-offs further constrain the practical translation of nutritional synergy into acceptable food products. Increasing the legume fraction may improve protein quality and lysine content, but may simultaneously intensify beany flavour, bitterness, astringency, darker colour, gritty mouthfeel, or reduced product expansion in extruded snacks [37]. Similarly, fermentation may enhance flavour complexity and reduce antinutritional factors, but excessive acidification may reduce acceptability among consumers who prefer milder flavour profiles. Thus, nutritional optimisation must be balanced with texture, flavour, colour, aroma, cultural familiarity, and affordability for cereal–legume products to achieve sustained consumer adoption.

Antinutrient–phenolic interactions also complicate the interpretation of synergy. While reductions in phytate and tannins can improve mineral availability and protein digestibility, phenolic compounds may simultaneously contribute antioxidant and anti-inflammatory properties [4]. Excessive removal or degradation of these compounds may, therefore, improve one nutritional endpoint while weakening another. In addition, phenolic–protein and phenolic–starch complexes may either protect bioactives during digestion or reduce protein digestibility and starch hydrolysis, depending on molecular structure, concentration, and processing history [38]. These complexities indicate that cereal–legume functionality is governed by balancing effects rather than simple additive improvements.

Inadequate processing parameters are among the most important factors limiting the expected benefits of cereal–legume systems. Mild fermentation or germination may reduce phytate, tannins, and enzyme inhibitors, thereby improving mineral bioaccessibility and protein digestibility; however, uncontrolled fermentation, excessive germination, overheating, over-roasting, or severe extrusion may produce undesirable outcomes. These may include lysine loss through Maillard reactions, protein aggregation, starch over-gelatinisation, degradation of heat-sensitive bioactive compounds, and the formation of dense matrix structures that restrict enzyme access during digestion [39]. Therefore, processing should not be regarded as inherently beneficial, but rather as a controllable intervention, the outcomes of which depend on careful optimisation.

Although cereal–legume combinations are frequently presented as nutritionally and technologically synergistic systems, the available evidence is not uniformly positive and should therefore be interpreted with care. Synergy is strongly influenced by crop genotype, the cereal-to-legume ratio, particle size, moisture content, processing temperature, residence time, fermentation microbiota, germination duration, and storage conditions. Consequently, the same processing approach may improve nutrient bioaccessibility in one matrix while reducing protein quality, mineral availability, antioxidant activity, or sensory acceptability in another. This variability explains why several reported benefits remain context-dependent rather than universally reproducible.

## 5. Bioactive Compound Synergies in Cereal–Legume Systems

Cereal–legume food matrices function as integrated systems, with effects on metabolic health, bioactivity, and sensory performance that extend beyond protein complementation and macronutrient balance, driven by complex phytochemical–nutrient interactions. Both groups are rich in phenolic acids, flavonoids, tannins, and isoflavones, which exhibit antioxidant, anti-inflammatory, and glycaemic regulatory activities [1]. In composite foods, these substances act synergistically rather than independently, interacting with starch, proteins, non-starch polysaccharides, minerals, and other phytochemicals to optimise matrix design and physiology [40]. Phenolic compounds in cereals are predominantly present in bound forms, linked to cell wall components such as arabinoxylans and lignin within the bran layers, whereas legumes generally contain a higher proportion of soluble phenolic compounds, including flavonoids and isoflavones, which are primarily localised in vacuoles and, to a lesser extent, associated with protein bodies [41]. Their concomitant formation yields a phenolic profile that is complementary to the former; the combination of bound hydroxycinnamic acid (e.g., ferulic acid) with soluble compounds such as genistein and daidzein improves redox cycling, radical stabilisation and enzyme inhibition beyond additive actions [4]. Many phenolics also interact with macronutrients. Protein–phenolic interactions influence digestibility, enzyme access, and the release of bioactive peptides. Changes in gelatinisation, retrogradation, and enzymatic hydrolysis with phenolic–starch complexation flavour lower postprandial glycaemic responses [23]. Depending on the structure and concentration of a phenolic compound, phenolic–mineral chelation may either reduce mineral bioavailability or increase mineral bioavailability. Processing technologies help to modulate these interactions. Fermentation releases bound phenolics and increases isoflavone aglycones; germination enhances phenolic content, while extrusion and roasting improve accessibility through structural disruption and Maillard-derived antioxidants [39,42]. Overall, cereal–legume matrices represent integrated phytochemical ecosystems with structural interactions and processing histories that dynamically regulate nutrient–bioactive synergies.

### 5.1. Phenolic Profiles of Cereals and Legumes

#### 5.1.1. Phenolic Composition of Cereals

Cereals such as wheat, sorghum, millet, maize, barley, and oats are the main sources of dietary phenolics; these are predominantly derived from phenolic acids, especially hydroxycinnamic acids such as ferulic, p-coumaric, caffeic, and sinapic acids. Ferulic acid is a common phenolic in cereal brans and has been reported to form a large proportion of overall phenolics found in wheat and maize. These compounds mainly occur in bound forms and are esterified or ether-linked to related species, such as arabinoxylans, hemicelluloses, and lignin, in cell wall matrices, especially in the aleurone and bran layers, with low native extractability [39]. Certain cereals, such as sorghum, have also been characterised as having specific bioactives (3-deoxyanthocyanidins and condensed tannins), with potent antioxidant, anti-inflammatory, and carbohydrate-digestive enzyme-inhibitory activities [43]. Cereal phenolics are predominantly present in bound forms and, therefore, exhibit limited bioaccessibility in the upper gastrointestinal tract. Their physiological benefits are enhanced through processing interventions such as fermentation, germination, and extrusion, which promote the release of bound phenolics. In addition, colonic microbial hydrolysis further releases phenolic compounds from the food matrix, increasing their bioavailability and enhancing biological activity.

#### 5.1.2. Phenolic Composition of Legumes

Legumes, including soybean, chickpea, lentil, cowpea, pigeon pea, and Bambara groundnut, exhibit a variety of rich phenolic profiles that are more diverse than those found in most cereals. In contrast to cereals, where phenolic compounds are primarily attached to cell walls, legumes have a greater amount of free and soluble phenolics. This characteristic enhances their extractability and bioaccessibility [41]. Major constituents include flavonoids (quercetin and kaempferol), isoflavones (genistein, daidzein, and glycitein), which are particularly abundant in soybeans, and condensed tannins concentrated in coloured seed coats.

Isoflavones are unique phenolic compounds found in legumes, primarily appearing as glycosides that transform into more active aglycones during processing or digestion. These compounds are commonly linked to various health benefits, including cardioprotection, antioxidant properties, anti-inflammatory effects, and phytoestrogenic activity, all of which help prevent chronic diseases and promote metabolic health [44]. Additionally, flavonoids and tannins from legumes demonstrate significant abilities to scavenge radicals and inhibit carbohydrate-digestive enzymes. Generally, phenolics are located in vacuoles or protein bodies rather than firmly attached to cell walls, which facilitates their release and absorption in the intestines. Nonetheless, the interactions between phenolics and proteins can affect both the digestibility of proteins and the bioaccessibility of phenolic compounds.

#### 5.1.3. Bound and Free Phenolics: Complementarity in Composite Systems

**Figure 2:**
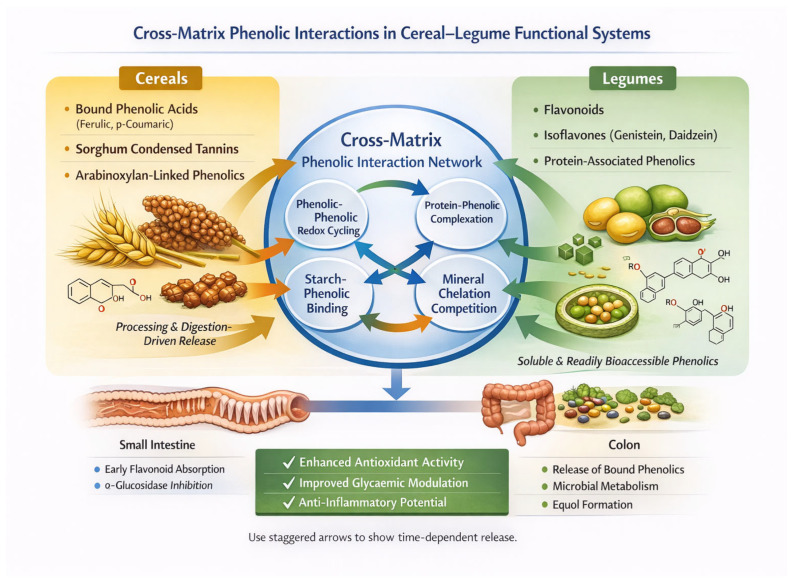
Synergistic antioxidant effects.

Cereal–legume composite foods have attracted considerable attention as sustainable dietary strategies to improve nutritional quality, enhance food security, and promote health benefits. Owing to their distinct bioactive profiles (Table 3), cereals and legumes form a complementary phenolic spectrum when combined in food systems. Cereals are particularly rich in bound phenolic acids, notably hydroxycinnamic acids such as ferulic and p-coumaric acids, which are associated with arabinoxylans and other cell wall components [39]. On the contrary, legumes have free and soluble phenolics, such as flavonoids and isoflavones, which are more extractable and bioaccessible [41]. Their composite broadens phenolic diversity through a better-integrated synthesis, potentially increasing antioxidant and metabolic activities beyond additive effects [4]. Cross-matrix interactions further enhance this complementarity and are shown in Figure 2. Phenolic–phenolic redox cycling can stabilise radicals, prolonging antioxidant activity [45].

Legume polyphenols also bind cereal proteins such as gluten and kafirin, influencing digestibility and functional properties. Phenolic–starch complexes with amylose can also reduce α-amylase and α-glucosidase hydrolysis, thereby enhancing glycaemic control [23].

### 5.2. Synergistic Antioxidant and Enzyme-Inhibitory Effects

Phenolic compounds exhibit antioxidant activity via hydrogen atom transfer, single-electron transfer, and metal chelation, leading to radical scavenging and reduced oxidative stress [55]. This antioxidant activity is influenced by structural properties such as the position of the hydroxyl group, degree of conjugation, and molecular configuration. Diverse subclasses of this type represent broad redox networks in cereal–legume composites; hydroxycinnamic acids from cereals and flavonoids or isoflavones from legumes, for example, generally lead to additive or synergistic antioxidant effects. Synergy occurs due to a variety of mechanisms. According to Shahidi and Ambigaipalan [55], phenolics are able to regenerate oxidised counterparts through redox cycling, which prolongs antioxidant activity. Structural diversity increases radical stabilisation by means of resonance effects and can facilitate scavenging activities. Similarly, protein and polysaccharide interactions help protect unstable phenolics, allowing continued activity during processing and digestion [38]. Fermented sorghum–cowpea blends have been proven to possess higher 2,2-Diphenyl-1-picrylhydrazyl (DPPH) scavenging and Ferric Reducing Antioxidant Power (FRAP) compared to single flours [56]. Fermentation releases bound cereal phenolics and promotes the production of legume aglycones, thereby fortifying cross-matrix antioxidant interactions.

#### 5.2.1. α-Amylase and α-Glucosidase Inhibition

Polyphenols help regulate postprandial glycaemic responses by inhibiting carbohydrate-digesting enzymes, notably α-amylase and α-glucosidase, which hydrolyse starch into glucose. Inhibition slows glucose release and reduces glycaemic excursions. Mechanisms include active-site binding, non-competitive conformational changes, and complexation with starch that limits enzyme accessibility [23]. Flavonoids and condensed tannins show strong affinity for these enzymes via hydrogen bonding and hydrophobic interactions with catalytic amino acids, reducing starch hydrolysis and glucose diffusion. McDougall and Stewart [49] reported that the magnitude of the inhibition relies upon the phenolic structure, particularly hydroxylation patterns and tannin polymerisation. To enhance a wider range of phenolic contents, bound cereal phenolic acids and soluble legume flavonoids, together with isoflavones, can be mixed to form cereal–legume composites which promote additive or synergistic enzyme inhibition, as reported by Shahidi and Ambigaipalan [55], whereas phenolic–starch complexes could further limit gelatinisation and enzyme access, resulting in delayed digestion [23]. According to Saini et al. [2], lentil–wheat and chickpea–barley blends exhibit stronger α-glucosidase inhibition and greater potential for superior glycaemic control than single grains.

#### 5.2.2. Anti-Inflammatory Potential

Phenolic compounds attenuate inflammatory signalling related to metabolic disorders and chronic low-grade inflammation. They modulate intracellular pathways including nuclear factor kappa B (NF-κB) and mitogen-activated protein kinase (MAPK), which, in turn, reduce the transcription of pro-inflammatory mediators such as tumour necrosis factor-α and interleukin-6. In cereal–legume composite systems, the synergistic role of complementary phenolics can augment anti-inflammatory properties. Legume isoflavones, such as genistein and daidzein, possess phytoestrogenic and anti-inflammatory activities by downregulating NF-κB and inhibiting cyclooxygenase-2 expression [44]. When used in conjunction with cereal phenolic acids, such as ferulic acid, more widespread synergistic effects could be observed [55]. Synergy also emerges from the suppression of oxidative stress. Hydroxycinnamic acids and flavonoids decrease reactive oxygen species, thus indirectly inhibiting NF-κB activation and cytokine production [55]. Phenolics further modulate the gut microbiota, producing anti-inflammatory metabolites and bioactive isoflavone derivatives [29]. Nevertheless, evidence of cereal–legume anti-inflammatory synergy in vivo is limited and requires further clinical validation.

### 5.3. Bioaccessibility and Bioavailability of Combined Bioactives

#### 5.3.1. Matrix-Driven Modulation of Bioaccessibility

Bioaccessibility is the proportion of a bioactive compound released from the food matrix during digestion and available for absorption. It is primarily determined by matrix structure and physicochemical interactions in cereal–legume composites. The networks of dietary fibre, proteins, starch granules, and phenolics can either promote or impede the release of bioactives [29]. Cereal dietary fibre, especially arabinoxylans and non-starch polysaccharides, can entrap soluble legume flavonoids, inhibiting their release in the small intestine and delaying their diffusion through the gut [39]. Legume and pigmented cereal tannins form protein–phenolic complexes through hydrogen bonding and hydrophobic interactions, modulating protein digestibility and phenolic availability [38]. Phenolics also chelate minerals, including iron, zinc, and calcium, which, in turn, can decrease mineral bioavailability but may also stabilise redox-active metals and improve antioxidant protection [55]. Polyphenol stability and release depend significantly on matrix complexity; hence, evaluating phenolics in structural and processing contexts is important.

#### 5.3.2. Phenolic Interactions Across Mixed Food Matrices

Interactions between phenolic compounds in cereal–legume composites remain underexplored despite their functional importance. In mixed matrices, phenolics take part in redox cycling, reforming oxidised molecules and stabilising radicals to extend antioxidant activity during processing and digestion [55]. Phenolics also form soluble or insoluble complexes with proteins through hydrogen bonding and hydrophobic interactions, which can affect protein conformation, enzymatic susceptibility, and phenolic stability, influencing digestibility and bioactive release [38]. Interactions between phenolics and starch alter gelatinisation and retrogradation by interfering with amylose recrystallisation, disrupting enzymatic hydrolysis, and affecting glycaemic response [23]. In the colon, bound cereal phenolics resist upper-gut digestion and are released by microbial esterases, while many soluble legume flavonoids are absorbed earlier, promoting multi-site antioxidant activity [39]. Isoflavones, e.g., daidzein, are metabolised by microbes into the bioactive metabolite, while phenolic acids are metabolised into smaller metabolites [44]. The combined phenolic fermentation pathways in cereal–legume composites remain poorly studied. This represents a critical mechanistic gap, as the interactions among phenolics, proteins, starches, and microbial metabolites likely determine the ultimate health functionality of these foods. Advanced in vivo bioavailability studies and omics-driven approaches are needed to clarify these interaction pathways.

#### 5.3.3. Processing-Driven Enhancement

Processing, including fermentation, germination, and extrusion, significantly influences the structural and functional properties of phenolics in cereal–legume composites. These processes drive enzymatic and physicochemical activity, facilitating the release and transformation of phenols and their interactions. Fermentation increases free phenolics through microbial esterases that hydrolyse bound cereal phenolics, including ferulic acid, and converts isoflavone glycosides into more bioactive aglycones with enhanced absorption [29,44]. Germination activates endogenous enzymes that soften the cell wall, facilitating the release of phenolics and enhancing antioxidant activity. It also decomposes phytates, improving mineral bioavailability and reducing chelation constraints [55]. Extrusion uses heat, pressure, and shear to disrupt cell structures, gelatinise starch, and denature proteins, thereby increasing phenolic accessibility. Although excessive heat may degrade sensitive compounds, optimised conditions improve the extractability of phenolics and antioxidant capacity, and reduce tannin–protein complexes, thereby improving digestibility [38]. These procedures enhance the bioaccessibility and techno-functional quality of cereals and legume food products.

## 6. Processing-Driven Modulation of Synergy

Processing in cereal–legume systems extends beyond preservation, acting as a biochemical and physicochemical modulator that reshapes nutrient structure, bioactive availability, and molecular interactions (Table 4). Health benefits of plant foods are strongly influenced by processing-induced changes in proteins, starches, fibres, and phytochemicals [55]. Thus, cereal–legume composites are dynamic systems where processing governs both composition and interaction networks.

Processing affects the release and transformation of phenolics, proteins, starches, and antinutrients. Fermentation and germination hydrolyse bound phenolics, increasing free phenolic acids and antioxidant activity [39]. Thermal treatments, such as extrusion, disrupt starch and denature proteins, alter the phenolic interactions that influence digestibility and glycaemic response [23]. Antinutrients, such as phytates, are also reduced, thereby improving mineral and protein bioavailability [22]. These transformations occur within integrated matrices where structural reorganisation modulates bioactive functionality.

### 6.1. Traditional and Emerging Processing Methods

**Figure 3:**
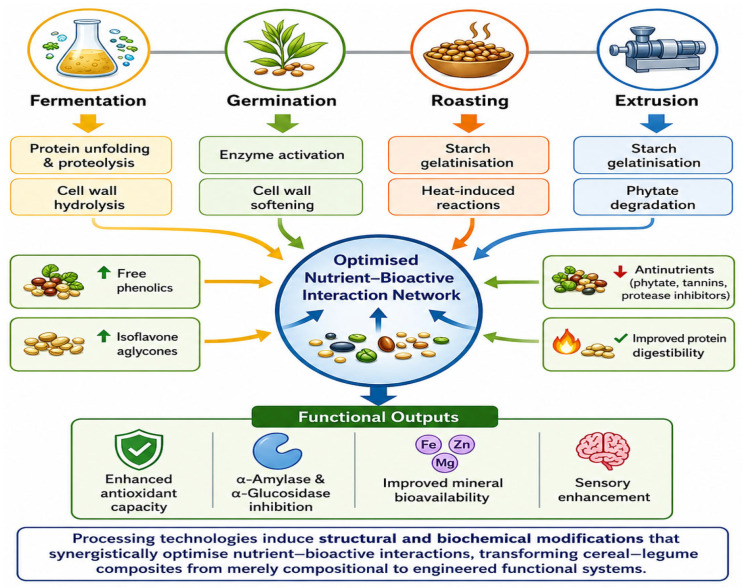
Processing-driven engineering of synergy in cereal–legume systems.

Traditional processing techniques such as fermentation, soaking, germination, and roasting have long been used to improve the nutritional quality, digestibility, and sensory properties of cereal–legume foods (Figure 3). Emerging technologies, including extrusion, enzymatic treatment, high-pressure processing, and pulsed electric fields, are increasingly applied to enhance nutrient bioavailability, functional properties, and the stability of bioactive compounds.

#### 6.1.1. Fermentation

Fermentation, especially lactic acid fermentation, is widely used in cereal–legume foods such as *ogi*, *injera*, and sourdough. Beyond preservation and flavour, it acts as a biochemical modulator that enhances nutrient interactions and phytochemical availability through enzymatic hydrolysis, acidification, and microbial metabolism [29].

Fermentation hydrolyses bind cereal phenolics via microbial esterases, releasing hydroxycinnamic acids, such as ferulic acid, thereby improving antioxidant capacity [39]. It also converts legume isoflavone glycosides into more bioactive aglycones, which are better absorbed [29]. Phytase activity degrades phytates, enhancing mineral bioavailability and modifying mineral–phenolic interactions [60], while proteolysis releases bioactive peptides with antioxidant and antihypertensive effects [61]. Fermentation further modifies protein–phenolic and starch–phenolic interactions, improving digestibility and enzyme inhibition, though optimisation is required to preserve phenolics and sensory quality [55].

#### 6.1.2. Germination/Malting

Germination or malting is a controlled biological process that activates endogenous hydrolytic enzymes, including amylases, proteases, and phytases, leading to substantial biochemical reorganisation within cereal–legume matrices. During germination, enzymatic softening of cell wall structures facilitates the release of phenolic compounds that were previously bound to structural polysaccharides. As a result, total phenolic content may increase due to both de novo synthesis and liberation from bound forms [39]. This enhanced availability of phenolics has been associated with improved antioxidant capacity and increased inhibitory activity against carbohydrate-digesting enzymes [62].

In addition to its effects on phenolics, germination promotes partial proteolysis, thereby improving protein digestibility and potentially leading to the formation of bioactive peptides with antioxidant or antihypertensive properties. At the same time, phytase activation promotes phytate degradation, thereby enhancing mineral bioavailability and reducing mineral chelation [22]. Germination can also reduce tannin content or modify tannin–protein interactions, further improving nutritional quality. Furthermore, partial starch hydrolysis during germination reduces paste viscosity in cereal–legume porridges, enabling higher-nutrient-density formulations, which is particularly beneficial in infant and complementary foods, while maintaining bioactive functionality.

#### 6.1.3. Roasting

Roasting is a dry-heat treatment that induces structural and chemical transformations in cereal–legume composites. Thermal exposure may partially degrade heat-sensitive flavonoids; however, moderate roasting conditions can increase extractable phenolic content by disrupting cell wall matrices and enhancing solvent accessibility [57]. Abdalla and Sumon [3] reported that roasting initiates Maillard reactions between reducing sugars and amino acids, generating Maillard reaction products (MRPs) that may possess antioxidant properties and contribute to overall radical-scavenging capacity.

From a sensory perspective, roasting enhances flavour, aroma, and colour development, thereby improving consumer acceptability of functional composite foods. Nevertheless, excessive thermal intensity may promote phenolic polymerisation, protein cross-linking, and structural rigidity, potentially reducing phenolic bioaccessibility and digestibility [38]. Therefore, roasting conditions must be carefully optimised to balance flavour enhancement with preservation of bioactive functionality.

#### 6.1.4. Extrusion and Composite Flour Production

Extrusion cooking is a rapid, high-temperature, short-term method used to apply heat, pressure, and mechanical shear to cereal–legume mixtures, leading to dramatic changes in their structure. Extrusion promotes starch gelatinisation, protein denaturation, and partial depolymerisation of dietary fibre, thereby altering phenolic–macronutrient interactions within the matrix. Starch gelatinisation may enhance phenolic accessibility, and protein unfolding exposes additional binding sites for phenolics, potentially promoting antioxidant interactions [23]. Moreover, extrusion can inactivate heat-labile antinutritional factors, enhancing protein digestibility and mineral availability. However, extrusion conditions, such as temperature, moisture content, and screw speed, significantly affect phenolic stability. Excessive heat or prolonged residence time have been reported to degrade some phenolic compounds, and optimised extrusion parameters are demonstrated to enhance total antioxidant activity and digestibility or sensory quality [23]. Interaction patterns are also related to the stage at which blending occurs in composite flour production. Pre-fermentation blending of cereals and legumes can facilitate cross-matrix enzymatic synergy during processing, supporting phenolic liberation and reorganisation mechanisms. However, post-process blending can inhibit interactive transformation and lead to more additive than synergistic functionality. Thus, the sequencing of processing and blending operations significantly contributes to the engineering of functional synergy in cereal–legume systems.

### 6.2. Effects on Nutrients, Bioactives, and Antinutrients

Processing in cereal–legume systems modulates the nutrient composition, structure, and their interactions, thereby affecting digestibility, bioactivity, and the balance of beneficial and antinutritional constituents. Protein digestibility is typically enhanced by denaturation and proteolysis, thereby improving enzyme access and amino acid availability [61]. Conversely, starch digestibility is limited by the formation of phenolic complexes with amylose or amylopectin, which reduce enzymatic hydrolysis and reduce the glycaemic response [23]. Mineral bioavailability increases with the hydrolysis of phytates, leading to decreased chelation of iron, zinc, and calcium [60]. Bioactive profiles are modified as well. Germination and fermentation enhance the levels of free phenolics and antioxidant potential, and legume isoflavone glycosides are converted to more bioactive aglycones [44]. As a result, antioxidant capacity and α-glucosidase inhibition often increase [4]. Phytates, tannins, and protease inhibitors are reduced during processing, allowing better utilisation of nutrients. Moderate amounts of these nutrients may still have a health-promoting effect and need to be optimised for maintenance rather than removed from plants.

### 6.3. Processing-Driven Optimisation of Synergistic Interactions in Food Matrices

Traditional cereal–legume studies focus on compositional enhancement of cereals, such as protein enrichment, but functional quality is also closely linked to matrix interactions and processing-driven transformations [4]. Processing should be interpreted as a technique to design synergistic networks of nutrients and bioactives. Fermentation, germination, extrusion, and controlled heating can enhance phenolic release without altering the structure. During fermentation, bound cereal phenolics are liberated, enhancing antioxidant activity without compromising heat-sensitive compounds [39]. Optimised extrusion enhances gelatinisation and unfolding of starch, improving access to and digestibility of phenolic compounds [23]. Processing also promotes protein–phenolic and starch–phenolic interactions that exert some moderation on carbohydrate digestion and glycaemic response [38]. Instead of reducing the levels of antinutrients, the reduction in phytate and tannin by optimisation is balanced and protective bioactivity is retained [4]. Overall, integrative optimisation frameworks assist in the design of health-promoting composite foods. Although processing is increasingly recognised as a tool for modulating synergistic nutrient–bioactive interactions, optimisation frameworks remain fragmented and largely empirical. There is insufficient comparative research establishing standardised processing windows that balance nutrient preservation, bioactive accessibility, glycaemic modulation, and sensory quality. Future work should move toward predictive and systems-based optimisation models for cereal–legume food design.

### 6.4. Strategic Framework for Synergy Optimisation in Cereal–Legume Matrices

For food technologists, synergy optimisation can be translated into a practical design framework built around four interconnected decisions: selection of an appropriate cereal–legume combination, determination of the optimal blend ratio, application of a suitable processing sequence, and adjustment of processing parameters to achieve the intended nutritional, functional, and sensory outcomes. Within this framework, cereals primarily contribute starch structure, expansion potential, mild flavour, and energy density, whereas legumes enhance lysine content, protein density, mineral contribution, emulsification, and bioactive potential [1] (Table 5). Thus, the objective extends beyond simply fortifying cereals with legumes; rather, it involves engineering an integrated food matrix in which processing strategically enhances bioactive release, reduces limiting antinutritional factors, and produces a sensory profile acceptable to consumers.

The practical application of this framework varies according to the intended product category. For complementary foods and smooth porridges, a cereal-to-legume ratio of approximately 70:30 to 60:40 may serve as a practical starting point, as this range generally improves protein quality and lysine balance while limiting excessive beany flavour, bitterness, and undesirable viscosity. To maximise these benefits, a processing sequence involving soaking, followed by germination or mild fermentation, drying, milling, and final cooking may be adopted. Germination for approximately 24–48 h under hygienic moist conditions can activate endogenous phytases and amylases, thereby supporting phytate degradation, improving mineral release, and enhancing partial starch modification [39]. Subsequent mild lactic fermentation for about 12–24 h at 30–37 °C may further reduce phytate, improve flavour complexity, and increase free phenolic availability; however, excessive fermentation should be avoided to prevent unacceptable sourness [29].

For ready-to-eat extruded snack products, the food design priorities shift towards structural expansion, crispness, and shelf stability. Accordingly, a cereal-to-legume ratio of approximately 75:25 to 65:35 may provide a suitable preliminary formulation range, allowing cereals to maintain expansion characteristics while legumes improve protein quality and nutritional density. A targeted processing pathway may include dehulling or soaking of legumes, optional short germination, drying, milling, blending, and extrusion cooking. Since extrusion strongly influences product structure and nutrient functionality, moderate moisture and controlled thermal-shear conditions are essential to balance expansion, crisp texture, and retention of heat-sensitive bioactives. As a practical starting point, feed moisture levels of approximately 14–20%, container temperatures of 120–160 °C, and short residence times may be explored prior to product-specific optimisation. Excessive heat or shear should be avoided because these conditions may reduce lysine availability, darken product colour, harden texture, and degrade sensitive phenolic compounds [23].

When the nutritional objective is glycaemic modulation, formulation strategies should prioritise slowly digestible starch, resistant starch, dietary fibre, and beneficial phenolic–starch interactions without introducing excessive bitterness or astringency. A practical approach may involve combining sorghum, millet, or maize with approximately 25–35% cowpea, Bambara groundnut, soybean, or common bean, followed by germination or fermentation prior to controlled cooking or extrusion. Giuntini and Sardá [62] reported that this strategy may generate a food matrix characterised by reduced phytate levels, improved protein digestibility, retained or liberated phenolics, moderated starch hydrolysis, acceptable aroma, and suitable textural characteristics depending on the format of the final product.

For high-protein composite flour systems, particularly those intended for porridges, dough systems, or bakery formulations rather than highly expanded snack products, higher legume inclusion levels of approximately 35–45% may be considered. However, increasing the legume content also intensifies sensory challenges, including beany aroma, bitterness, astringency, and gritty mouthfeel. Consequently, stronger sensory mitigation strategies such as dehulling, soaking, fermentation, roasting, or flavour balancing become necessary [37]. Under such circumstances, formulation decisions should not just be driven by nutrient composition but also by concurrent sensory optimisation and consumer acceptability testing.

Importantly, these formulation and processing windows should be regarded as starting points for optimisation rather than universal practices. The optimal design will ultimately depend on crop genotype, flour particle size, initial moisture content, antinutrient burden, the desired product format, consumer preferences, and processing scale. Nevertheless, this framework makes synergy optimisation more actionable by explicitly linking blend composition and processing design to measurable outcomes such as protein quality, mineral bioaccessibility, phenolic availability, glycaemic response, texture, flavour, and overall acceptability.

## 7. Sensory Synergy and Consumer Acceptability

### 7.1. Sensory Challenges of Cereals and Legumes

Despite their nutritional advantages, cereal–legume systems generally face sensory constraints that limit consumer acceptance. One of the most pressing issues is the strong, undesirable taste related to the bitterness, astringency, and ‘beany’ flavour of legumes, which are also included in these plant products. These sensory characteristic traits generally relate to bioactive compounds and lipid oxidation products. For example, phenolic compounds and tannins are believed to contribute to bitterness and astringency by interacting with salivary proteins, leading to precipitation and decreased lubrication in the oral cavity [4,65]. Much of the beany flavoured legumes, for example, cowpea, soybean, and Bambara groundnut, are due to lipoxygenase enzymes that catalyse the oxidation of polyunsaturated fatty acids into volatile compounds, including hexanal and pentanal [37]. Because these compounds are commonly classified as grassy, earthy, or rancid, they negatively impact product desirability. The phenolic compounds present in cereals, such as sorghum and millet, which reside in the bran layer, can give them a bitter taste and darker colour, reducing their palatability [1]. Issues with texture also prevail, especially in those containing legumes as the main ingredient. High protein content and fibre may result in dense, coarse, or gritty forms; a lack of gelatinisation in cereal ingredients for starch; and low cohesiveness and mouthfeel [66]. These sensory limitations highlight the importance of targeted formulation and processing interventions for acceptability.

### 7.2. Synergistic Sensory Balancing in Cereal–Legume Blends

The integration of cereals and legumes offers a distinct opportunity to enhance sensory experiences through their complementary interactions (Table 6). The generally mild flavours and starch-rich nature of cereal components can successfully dilute or conceal undesirable flavours associated with legumes. This effect, referred to as aroma masking, diminishes the perception of beany and bitter notes by reducing the concentration of volatile off-flavour compounds and altering their release during chewing [37].

Furthermore, various processing methods amplify this sensory synergy. For instance, fermentation is vital for refining flavour profiles as it generates appealing volatile compounds such as organic acids, alcohols, and esters while simultaneously breaking down compounds that contribute to off-flavours [67].

Similarly, roasting can induce Maillard reactions, generating flavour-active compounds that contribute to desirable roasted, nutty, and caramel-like notes, thereby masking inherent bitterness [23].

Improvements in texture and mouthfeel are also achieved through cereal–legume interactions. The starch-rich nature of cereals contributes to gel formation and viscosity, enhancing softness and cohesiveness in composite products such as porridges, breads, and extruded snacks. Meanwhile, legume proteins can improve water absorption and emulsification properties, contributing to better structure and mouthfeel when optimally balanced [66]. The formation of protein–starch networks during processing further enhances structural integrity and sensory appeal.

In extrusion processing, for instance, starch gelatinisation and protein denaturation lead to expanded structures with desirable crispness and reduced grittiness, while also improving flavour through thermal reactions [23]. Therefore, integrating cereals and legumes, combined with appropriate processing, enables the development of products with improved sensory profiles and consumer acceptability.

### 7.3. Relationship Between Bioactive Content and Sensory Perception

Bioactive compounds such as phenolics, flavonoids, and tannins contribute to both the health benefits of cereal–legume systems and their sensory properties. In addition to providing antioxidant, anti-inflammatory, and glycaemic-modulating properties, these substances are also responsible for sensory attributes such as bitterness, astringency, and colour changes [4]. Sensory perception is profoundly influenced by the interaction between bioactive and macronutrient compounds. Phenolic–protein interactions can yield complexes that decrease protein solubility and alter texture, as well as modify the release of flavour compounds [29]. Similarly, phenolic–starch interactions influence starch digestibility and glycaemic response but may also affect mouthfeel and viscosity. The processing of bioactive compounds helps manage the health and sensory implications of these substances. Fermentation and germination can enhance the release of bound phenolics and increase antioxidant activity while reducing bitterness through enzymatic modification [67]. Some phenolic compounds are susceptible to overprocessing, especially at high temperatures, which can lead to degradation and a reduction in health benefits while increasing sensory acceptability by reducing bitterness [23]. The trade-off between health and sensory quality is critical, as consumer perception is mediated by this balance. Moderate levels of bioactive compounds can enhance the product’s attractiveness by supporting functional claims and subtle flavour complexity, whereas excessive levels can lead to rejection due to severe bitterness or astringency. It is, therefore, important to achieve the correct concentration of bioactive compounds in cereal–legume food matrices through well-controlled processing and formulation, providing not only nutritional functionality but sensory acceptability as well.

### 7.4. Conceptual Model Linking Processing, Bioactive Modulation, and Sensory Acceptability

A clearer health–sensory model can be conceptualised in which processing parameters determine the extent of matrix disruption or restructuring; these matrix modifications subsequently regulate the release, transformation, and binding of phenolics, flavonoids, peptides, phytates, and tannins. These biochemical changes simultaneously influence health-related outcomes such as antioxidant potential, mineral bioavailability, protein digestibility, and glycaemic response, as well as sensory attributes including bitterness, astringency, aroma, colour, mouthfeel, and texture [4] (Table 7). Therefore, processing should be interpreted as a dual-function tool that can enhance nutritional functionality only when the resulting sensory profile remains acceptable to consumers.

Fermentation, germination, and extrusion illustrate this dual mechanism particularly well. Fermentation may increase free phenolics, degrade phytate, and reduce beany notes through microbial metabolism, but excessive acid production can generate an overly sour flavour [29].

Germination can activate endogenous enzymes that reduce antinutrients and improve digestibility, while also generating malty aromas and softer textures; however, prolonged germination may increase enzymatic browning, the development of a grassy flavour, and microbial risk [39]. Extrusion can reduce antinutrients, alter starch digestibility, and produce expanded, crispy products, but excessive temperature, shear, or residence time may degrade heat-sensitive bioactive compounds, intensify browning, and result in hard textures [23]. These examples demonstrate that the translational value of cereal–legume products depends on the identification of processing gaps that maximise bioactive benefits while maintaining desirable sensory attributes.

Accordingly, the central optimisation question is not whether processing improves cereal–legume matrices in general, but rather which processing conditions produce the most favourable combination of bioactive retention or release, antinutrient reduction, texture development, flavour improvement, and consumer acceptability. This dual health–sensory framework strengthens product development by linking laboratory indicators of nutritional improvement with practical market adoption.

## 8. Health Implications of Cereal–Legume Synergy

### 8.1. Matrix-Mediated Modulation of Glycaemic Response and Metabolic Health

Cereal–legume combinations influence metabolism through collaborative effects of nutrients, including protein quality and health benefits, as well as through the food matrix’s impact on starch digestibility and glucose dynamics [36]. Generally, cereal grains contain a significant amount of rapidly digestible starch, leading to elevated postprandial glucose levels [68]. In contrast, legumes have resistant starch, slowly digestible starch fractions, viscous dietary fibre, and protein networks that slow starch digestion when mixed with composite foods [64]. These mechanisms account for the lower digestibility of starch. The proteins and soluble fibres of legumes form a barrier coating on cereal starch granules, isolating the starch and lowering its amylase enzyme degradation, hence reducing apparent or total starch content, and reducing effective starch availability [69]. Fermentation has also been reported to increase resistant starch (RS) content in cereal foods, as the organic acids produced during fermentation may also reduce amylase activity [70,71]. Increased dietary fibre levels also increase intestinal viscosity and inhibit enzyme access to starch, thereby decreasing the glycaemic index (GI) of cereal foods [63]. But these protective effects can become compromised by high levels of heat, such as the extrusion process, which denatures native structural matrices and causes early gelatinisation of starch, reduces enzyme inhibitors, and with it yields greater apparent starch content than in the case of milder processes, such as germination and fermentation [72]. Nevertheless, the reported success of cereal–legume diets in reducing the GI of foods appears highly variable across the literature, due to varying proportions of cereal–legume ratios, fibre breakdown, particle size, and processing intensity [73].

In particular, the metabolic benefits of cereal–legume composite foods are not universal, as their GI effects are highly dependent on the manufacturing process that appears to confer those benefits [74]. Matrix structural modifications that affect processing conditions can positively or negatively affect the extent of benefits. Metabolically, matrix structure-induced alterations may be clinically relevant for the management of metabolic pathways underlying insulin resistance, obesity, and type 2 diabetes mellitus, through improved glycaemia and reduced postprandial plasma glucose levels [75,76]. However, this approach has the limitation that most available data are based on in vitro digestion models or studies of isolated ingredients rather than whole cereal–legume food systems. This reliance on in vitro studies may, however, overestimate physiological benefits, because these models cannot fully capture the complexity of human digestion, hormonal regulation, and inter-individual metabolic variability. As a result, claims regarding glycaemic modulation should be interpreted with reasonable caution and rigorously appraised only by well-designed human clinical intervention studies involving realistic food formulations. One major limitation is that much of the evidence comes from studies using isolated ingredients rather than intact food matrices, and from overreliance on in vitro models that may overestimate effects. The benefits described are therefore context-dependent, based on variability in processing, cereal–legume ratios, and study design, and human clinical evidence is also limited, with translation to real-world dietary outcomes being uncertain.

### 8.2. Modulation of Gut Microbiota and Short-Chain Fatty Acid Synthesis

Cereal–legume systems have been demonstrated to modify metabolic health via microbiota-driven mechanisms, including the anaerobic fermentation of non-digestible carbohydrates in the colon [77]. Cereals and pulses contain multiple fermentable substrates, including resistant starch, arabinoxylans, glucans, and oligosaccharides, which resist digestion in the upper gastrointestinal tract and are subsequently used by the microbiota in the colon to produce short-chain fatty acids (SCFAs), specifically acetate, propionate and butyrate [78,79]. SCFAs are important mediators of host metabolism. Colonocytes derive most energy from butyrate and maintain the integrity of the intestinal barrier [80]. Propionate has an impact on hepatic gluconeogenesis and contributes to central appetite regulation [81]. According to Beane et al. [82], acetate exerts effects in the whole-body regulation of metabolic pathways and lipid metabolism. The cereal–legume matrix itself also supports substrates that = support a complex microbiota community; this has been linked to the enrichment of potential beneficial microbiota such as *Bifidobacteria* and *Lactobacilli*, and taxa linked to the production of the SCFA butyrate such as *Faecalibacterium prausnitzii* [83,84].

Processing significantly affects the availability of these fermentable substrates. Fermentation can, for example, enhance the levels of prebiotic oligosaccharides and the fermentation of complex carbohydrate structures into more digestible forms [85].

Germination can influence the solubility of arabinoxylan and the amount of available fibre, thereby affecting short-chain fatty acid (SCFA) production. However, specific processing techniques, such as high-temperature extrusion, can strongly modify the structure and fermentability of fibre with consequent differences in microbial fermentation mechanisms and SCFA composition. Consequently, cereals and legumes exhibit the capability to be modulated by the microbiota, depending on processing techniques. Most of these claims are supported by in vitro fermentation and animal research.

Since human microbiome responses are highly individualised, predicting the impact of different cereal–legume combinations on humans is challenging. While there are promising signs pointing toward modulation of the gut microbiota, much of this evidence is experimental. Such studies, however, are mainly in vitro and focused on animal studies, providing mechanistic insights; there is still uncertainty about how gut microbes respond in humans, a limitation called translational certainty, and this is a major research gap. Human microbiotas are highly individualised and influenced by host genetics, dietary habits, and lifestyle, meaning they are challenging to generalise. As such, future research should include standardised human intervention studies to assess the functional relevance of cereal–legume matrices in real dietary settings through microbiome sequencing and metabolomic analyses. Inter-individual variability may limit generalisability, and thus, standardised human intervention studies are required.

### 8.3. Synergies of Bioactive Compounds and Their Release

Cereal–legume matrices may enhance bioactive compounds during processing-induced interactions, such as polyphenols and bioactive peptides [86]. Abioye and Babarinde [87] found that interactions between legume polyphenols and cereal phenolics, like brown finger millet with an up to 10 mg/g phenolic content, which is relatively high, could change the behaviour of the final food matrix. Additionally, it could affect the antioxidant activity by promoting the formation of polyphenol–protein and polyphenol–starch complexes [88]. However, complexing could be accompanied by a functional compromise. Such polyphenol complexation in the food matrix with protein and fibre, which usually improves stability of antioxidants, can adversely affect bioavailability by reducing intestinal absorption [89]. Therefore, the processing technique would need to be appropriately used to improve its nutritional availability. Sprouting produced up to 67% bioaccessible phenolics in finger millets, while pressure cooking led to depletion of about 30–35% of extractable phenolic content [90]. But the amount of change varies widely depending on the level of processing, the type of grain used, and the matrix composition.

The observed improvement in bioactivity released via fermentation can be attributed to the breakdown of antinutrients such as phytates and tannins, and the release of phenolics bound in the cell wall from the cereal matrix, thereby increasing their availability at receptor sites for antioxidant activity and mineral bioavailability [91,92]. Particularly, fermentation occurs at much lower temperatures than in other cereal processing methods, e.g., extrusion, where certain polyphenols may not be preserved [93]. On the other hand, processing can enhance both the release of phenolic compounds and the breakdown of complexing agents, underscoring an essential equilibrium between the stability of bioactive substances and their bioavailability. However, existing evidence is variable, primarily derived from laboratory settings, and further validation is necessary to confirm its relevance to commercial food products. This highlights a broader translational gap in cereal–legume bioactive research, in which mechanistic findings from controlled laboratory studies have not been adequately tested in realistic food systems or human populations. Future studies should integrate assessments of food matrix complexity, processing variability, and bioavailability under physiological conditions to strengthen functional claims.

### 8.4. Processing-Sensory Synergies and Acceptability

Cereal–legume products offer numerous nutritional advantages, but their consumption is often limited by consumer preferences due to sensory challenges, such as undesirable beany flavours and dense textures [94]. Certain processing methods can mitigate these issues without compromising the products’ functional properties. For example, fermentation has been found to significantly enhance sensory attributes by reducing antinutritional factors and volatile compounds responsible for off-flavours (α-galactosides), leading to flavour profiles enriched with organic and lactic acids [95]. Study indicates that techniques such as soaking, sprouting, and fermenting millet–Bambara groundnut mixtures can lead to notable improvements in sensory evaluations regarding aroma, appearance, taste, texture, and overall acceptability compared to unprocessed samples [96]. Nevertheless, the effects on sensory characteristics may vary across formulations and are likely influenced by specific fermentation conditions and cereal-to-legume ratios.

In a similar vein, germination enhances the activity of endogenous enzymes, resulting in softer textures, modifications in starch structure, and reduced bitterness through the breakdown of phenolic compounds. This process could potentially make the products more appealing to consumers [97,98]. Extrusion technology may also enhance consumer acceptance by improving mouthfeel through an expanded product structure [99]. However, excessive use of thermomechanical energy during extrusion can compromise essential flavour compounds and functional bioactive components unless moisture levels and temperatures are carefully controlled [100,101]. These findings suggest that processing techniques should be carefully selected to enhance sensory attributes while preserving nutritional integrity. Overall, improvements in sensory qualities are highly context-dependent; furthermore, there is a scarcity of research directly linking processing methods to both sensory experiences and functional results, which limits predictability regarding consumer acceptance. Additionally, most studies assess sensory acceptability independently of nutritional or metabolic functionality, creating a disconnect between technological optimisation and consumer-relevant health outcomes. Future investigations should adopt integrated frameworks that simultaneously evaluate sensory perception, bioactive retention, and physiological functionality.

### 8.5. Implications for Non-Communicable Disease Prevention

Cereals and pulses can also be effectively blended to prevent diet-related non-communicable diseases [2]. They accomplish this by reducing glycaemic responses, modifying gut microbiota SCFA signals, and enriching the diet with antioxidant polyphenols, thereby impacting various metabolic pathways associated with diabetes, cardiovascular disease, and obesity [86,102]. But these health effects differ across blends and depend on the cereal–legume ratio, processing technique, and overall diet context. Although there is growing evidence regarding an inverse association between healthy dietary patterns and metabolic syndrome, consumption of cereal and leguminous foods can be beneficial due to their high dietary fibre and bioactive compound content [103,104]. As cereals and legumes may be processed into food products to realise their full value, it is necessary to modify processing methods to overcome processing inconsistencies and sensory limitations. It is possible to use controlled germination and fermentation to balance metabolic functionality with consumer appeal, rather than extensive food heating. The functionality of cereal–legume products is derived not only from composition but also from changes to structure induced by processing. A mechanistic rationale is provided, but the majority of evidence is observational or in vitro; evidence of clinical relevance and metabolic effects in actual populations still needs to be confirmed in human trials.

## 9. Sustainability and Food System Perspective

### 9.1. Climate-Resilient Production and Soil Health

The advantages of cereal–legume systems in agricultural and ecological settings may be agronomic; however, their effects on sustainable intensification are context-dependent. From an input perspective, the ability of legumes to fix nitrogen through rhizobia under diverse conditions may be promising to form a low-cost/efficient source of nitrogen (N) to the crop [3,4]. Fixation values of 50–200 kg N/ha/yr have been reported, but this obviously differs widely with species, soil type, and other limiting conditions and, hence, might not be practical during fieldwork [105,106,107]. Such systems can theoretically reduce greenhouse gas (GHG) emissions and minimise input costs by using less energy-intensive inorganic nitrogen fertiliser [108]. However, unstable establishment of legumes on-farm has also been linked to low and unreliable nitrogen fixation in low-input systems in sub-Saharan Africa due to the limited availability of rhizobia inoculants and constraints on system management.

In addition, legumes not only contribute nitrogen through fixation to enhance yield potential, but their residues also improve soil organic matter, microbial activity, nutrient availability, and soil structure, making legume–cereal cropping systems suitable for marginal environments [109]. Cereals, including millet, are commonly cultivated alongside legumes and are recognised for their resilience to drought and high temperatures [110]. Consequently, it has been proposed that specific cereal–legume farming systems may be effective in regions vulnerable to climate change [111]. Nonetheless, the evidence supporting this resilience must be supported through additional trials and long-term research. Although these claims show promise, they primarily depend on a limited number of field studies; thus, there is insufficient evidence of their widespread applicability across various agricultural settings.

### 9.2. Food Security and Dietary Diversification

Cereal–legume combinations have the potential to enhance food security and improve dietary quality in developing nations. However, factors such as availability, consumer preferences, and acceptability often diminish their effectiveness, thereby limiting their actual impact [111]. The proteins from cereals and legumes complement each other by supplying essential amino acids, including lysine and sulphur-containing amino acids. Thus, incorporating legumes into cereal-based diets may enhance the overall nutritional quality of these meals [86].

Nevertheless, the bioavailability of amino acids in cereal–legume diets can be influenced by various factors such as processing methods, antinutritional components, and cooking techniques. This creates a gap between theoretical and practical values regarding dietary quality, a critical aspect that is sometimes overlooked. In addition to amino acids, cereal–legume foods are sources of various micronutrients and beneficial substances, including calcium, iron, zinc, magnesium, and dietary fibre. However, variability in absorption can limit their capacity to enhance vitamin and mineral content.

Amid growing sustainability concerns regarding animal protein consumption, there is a push to reduce reliance on animal-sourced proteins. Nonetheless, cereal–legume foods may not fully substitute for the diverse range of nutrients provided by animal products due to issues related to digestibility and nutrient bioavailability.

More importantly, combining legumes with cereal-based foods promotes diversification and increases the balanced intake of macro- and micronutrients, which can help improve nutrition in modern diets characterised by heavily refined cereals. Innovative processing techniques, such as germination, fermentation, and extrusion, can enhance the nutritional value and sensory attributes of these food products. However, it is important to note that the effectiveness of cereal–legume combinations varies across specific contexts, including processing methods and targeted food systems; thus, they may not be universally applicable. It is conceivable that translating these innovations into new and acceptable products will require a multidisciplinary science encompassing food chemistry, nutrition, microbiology, and food engineering. However, collaborative research remains limited, impeding translation from lab to scalable products. The nutritional potential is clear, but evidence for consistent human benefits is limited, and practical implementation is constrained by variability in processing, bioavailability, and consumer acceptance.

## 10. Research Gaps and Interdisciplinary Needs

Despite the encouraging mechanistic understanding and noted advantages of cereal–legume products, significant knowledge gaps persist in their application. Table 8 outlines the primary deficiencies in processing, matrix interactions, clinical validation, and the integration of sensory attributes with health outcomes, along with suggested research needs and practical implications. This highlights areas where further work is required to translate mechanistic understanding into functional, consumer-acceptable food products.

Therefore, it is necessary to bridge research gaps through integrative and interdisciplinary approaches that link the process, matrix, nutrient bioaccessibility/bioavailability, metabolic effects, and interactions with the microbiome and sensory perception. In the absence of such research, the effectiveness of cereal–legume functional food innovations is speculative.

## 11. Conclusions

The study highlights the transformative potential of cereal–legume food matrices as functional systems to tackle current nutritional, health-related, and sustainability challenges. Extending traditional reductionist perspectives considers the effects on crop composition, and a summary of existing evidence confirms that the real value of cereal–legume systems lies in their behaviour within complex food matrices. And these protein–starch–phenolic networks interact critically to dictate nutrient bioavailability, functional performance, and physiological responses. The complementary nutritional characteristics of cereals and legumes, including amino acid balance, dietary fibre, and micronutrient content, form a strong basis for the design of nutritionally enriched food products.

The review demonstrates that processing technologies, such as fermentation, germination, roasting, and extrusion, exploit the full potential of systems. These mechanisms serve as synergy drivers regulating molecular structures, diminishing antinutritional factors, releasing and providing the accessibility of bioactive ingredients, and enhancing digestibility. Importantly, a properly tuned processing environment can enhance antioxidant activity and simultaneously modulate glycaemic response, while retaining or even improving product quality.

From a sensory perspective, the study shows that incorporating cereals and legumes is an effective intervention to overcome inherent sensory limitations, such as bitterness, astringency, and even beany flavour. Bad qualities can be managed or limitations reduced, and positive qualities, such as enhanced aroma, texture, and mouthfeel, are achieved by additive mixing and processing. Striking this balance between nutritional functionality and sensory acceptability is essential for both consumer adoption and the market sales of composite food products. Also, it is noteworthy that the balance between bioactivity and sensory perception shows the importance of optimisation for sensory attributes that may reduce acceptability if not handled well; bioactive compounds, when used in moderation, confer health benefits. This dual nature underscores the need for systems-based approaches that involve food chemistry, processing science, and sensory evaluation.

Overall, cereal–legume food matrices provide opportunities for the development of functional, affordable, sustainable food products, particularly in underutilised crop-dependent regions. Further studies should focus on advanced characterisation of matrix interactions, optimisation of processing parameters, and consumer-focused product development. Developing such strategies will enable scientific evidence to be translated into scalable, food-based innovations that advance public health, improve food security, and strengthen agri-food system resilience. The practical implication is that synergy optimisation should be applied as a formulation-processing decision tool where food technologists select the cereal–legume pair, choose an appropriate blend ratio, apply a targeted sequence such as germination-before-extrusion or fermentation-before-milling, and then optimise time, temperature, moisture, and shear to achieve a defined endpoint such as improved mineral bioaccessibility, lower glycaemic response, enhanced protein quality, or high consumer acceptability.

## Figures and Tables

**Table 1:** Inclusion and exclusion criteria for the screening and selection of relevant studies.

Screening Domain	Inclusion Criteria	Exclusion Criteria
Publication type	Peer-reviewed journal articles, reviews, and relevant foundational studies published mainly from 2000 to 2026.	Non-scientific reports, duplicated records, inaccessible full texts, and sources without sufficient methodological detail.
Food system focus	Studies on cereals, legumes, or cereal–legume composite matrices, especially those relevant to sub-Saharan African diets and product development.	Studies focused only on unrelated food groups or single raw materials without relevance to composite interactions.
Processing focus	Studies evaluating soaking, dehulling, germination, fermentation, roasting, cooking, parboiling, extrusion, or emerging processing technologies.	Studies with no processing component or no link between processing and nutritional, functional, or sensory outcomes.
Outcome focus	Evidence on nutrient bioavailability, protein quality, antinutrients, bioactive compounds, antioxidant activity, functional properties, sensory quality, or consumer acceptance.	Studies that reported composition only without interpretation of matrix effects, processing response, or relevance to food applications.
Language and relevance	English-language publications with clear relevance to food science, nutrition, sensory quality, or sustainable food systems.	Articles outside the review scope or lacking clear relevance to cereal–legume food matrices.

**Table 2:** Synergistic nutritional, functional, and agricultural benefits of indigenous cereal–legume systems.

Aspect	Cereals	Legumes	Synergistic Benefits	Reference
Nutritional	High in carbohydrates and energy; moderate protein; limited lysine	High in protein; rich in lysine and other essential amino acids; vitamins and minerals	Complementary amino acid profiles; improved protein quality and digestibility; enhanced micronutrient intake	[25,26]
Functional/Food Processing	Starch properties influence viscosity, texture, and structure in foods	Proteins provide emulsification, foaming, water/oil-binding, and gel formation	Improved texture, stability, and sensory attributes in composite foods (porridges, snacks, baked goods, and beverages)	[27,28]
Health and Bioactive Compounds	Dietary fibre; antioxidants; phenolic compounds	Dietary fibre; resistant starch; flavonoids; isoflavones	Enhanced antioxidant and fibre content; potential for functional/fortified foods addressing malnutrition and chronic diseases	[4,29]
Agricultural/Ecological	Adaptable to drought, poor soils, and diverse agroecological conditions	Nitrogen fixation improves soil fertility; resilient in marginal soils	Sustainable farming systems; reduced fertiliser dependence; improved soil health; supports crop rotation and intercropping	[30,31]
Cultural/ Dietary	A staple energy source in traditional diets	Often consumed in combination with cereals in traditional dishes	Culturally acceptable, nutritionally balanced, and widely integrated into traditional and modern food systems	[26,32]

**Table 3:** Comparative phenolic profiles and synergistic interactions in cereal–legume systems.

Component	Cereals	Legumes	Interaction in Composite Matrix	Functional Result	References
Phenolic form	Predominantly bound phenolics (ferulic and p-coumaric acids) linked to cell wall arabinoxylans	Higher proportion of free and soluble phenolics	Bound phenolics released during processing interact with soluble flavonoids	Enhanced total phenolic diversity	[39,46]
Flavonoids	Limited (except sorghum anthocyanins)	Quercetin, kaempferol, and catechins	Redox cycling between phenolic acids and flavonoids	Improved radical scavenging	[47,48]
Tannins	Present in sorghum (condensed tannins)	Condensed tannins in seed coats	Protein–tannin–starch complex formation	Reduced glycaemic response	[49,50]
Isoflavones	Rare	Genistein and daidzein (soybean)	Synergy with cereal phenolic acids	Anti-inflammatory modulation	[44,51]
Matrix binding	Cell wall ester linkage	Protein–phenolic association	Competitive/cooperative binding	Modulated bioaccessibility	[29,52]
Digestive release pattern	Released mainly in the colon	Released in the small intestine	Multi-target antioxidant protection	Sustained antioxidant activity	[53,54]

**Table 4:** Processing-driven modulation of nutrient–bioactive synergy in cereal–legume systems.

Processing Method	Structural Modifications	Effects on Bioactives	Effects on Antinutrients	Synergistic Impact	References
Fermentation	Ester hydrolysis; proteolysis; acidification	Increase in free phenolics; increase in isoflavone aglycones	Decrease in phytate; decrease in tannins	Enhanced antioxidant and enzyme inhibition	[4,29]
Germination/Malting	Enzyme activation; cell wall softening	Increase in total phenolic content; increase in bioaccessibility	Decrease in phytates; decrease in protease inhibitors	Improved digestibility and bioactive release	[22,39]
Roasting	Maillard reaction; partial denaturation	Increase in extractable phenolics (moderate heat)	Decrease in heat-labile inhibitors	Improved flavour and antioxidant activity	[57,58]
Extrusion	Starch gelatinisation; protein unfolding	Increase in phenolic accessibility (optimised conditions)	Decrease in trypsin inhibitors	Enhanced glycaemic modulation	[23,59]
Composite Flour Blending	Cross-matrix interaction	Phenolic–starch–protein complex formation	Controlled reduction	Optimised functional synergy	[29,52]

**Table 5:** Cereal–legume processing optimisation for desired functional outcomes.

Desired Outcome	Suggested Cereal–Legume Ratio	Recommended Sequence	Indicative Parameter Range	Expected Trade-off to Manage	References
Smooth complementary porridge with improved protein quality	70:30 to 60:40	Soaking, germination or mild fermentation, drying, milling, and cooking	Germination: 24–48 h; fermentation: 12–24 h at about 30–37 °C	Avoid excessive sourness, high viscosity, and beany flavour	[22,29]
Crisp ready-to-eat extruded snack	75:25 to 65:35	Legume dehulling or soaking, drying, milling, blending, and extrusion	Feed moisture: about 14–20%; barrel temperature: about 120–160 °C; short residence time	Avoid low expansion, hard texture, dark colour, and lysine loss	[23,59]
Glycaemic-control-oriented flour or snack	75:25 to 65:35	Germination or fermentation followed by controlled cooking or extrusion	Moderate heat and moisture to retain phenolic–starch interactions	Balance slower starch digestion with acceptable bitterness and texture	[63,64]
High-protein composite flour	65:35 to 55:45	Soaking or dehulling, fermentation or roasting, drying, and milling	Mild processing sufficient to reduce antinutrients and off-flavours	Higher legume content may increase beany aroma, astringency, and gritty mouthfeel	[24,37]

**Table 6:** Sensory challenges and synergistic solutions in cereal–legume systems.

Sensory Challenge	Source/Compound	Effect on Product	Synergistic/Processing Solution	Outcome	References
Bitterness	Phenolics and tannins	Unpleasant taste	Blending with cereals; fermentation	Reduced bitterness; improved flavour	[4,65]
Astringency	Polyphenol–protein interaction	Dry mouthfeel	Germination; enzymatic modification	Improved palatability	[29,65]
Beany flavour	Lipoxygenase activity (hexanal)	Off-flavour	Roasting; fermentation, extrusion	Aroma masking; flavour enhancement	[23,37]
Gritty texture	High fibre/protein	Poor mouthfeel	Starch gelatinisation; extrusion	Smooth texture	[23,66]
Dense structure	High legume content	Reduced acceptability	Optimised cereal–legume ratio	Improved softness and cohesiveness	[1,66]

**Table 7:** Processing effects on bioactives, health outcomes, sensory changes, and optimisation challenges.

Processing Technique	Processing Parameters	Bioactive/Antinutrient Modulation	Expected Health-Related Outcome	Sensory Change	Optimisation Concern	References
Fermentation	Starter culture or natural microbiota, time, temperature, pH, and moisture	Increases free phenolics and bioactive peptides; reduces phytate, tannins, and beany volatile compounds	Improved mineral bioavailability, protein digestibility, and antioxidant potential	Reduced beany flavour; improved sour aroma and flavour complexity	Excessive fermentation may create an overly acidic taste, soft texture, or inconsistent product quality	[29,39]
Germination	Soaking time, germination duration, temperature, humidity, and drying conditions	Activates amylases, proteases, and phytases; reduces phytate and enzyme inhibitors; may increase some phenolics	Improved digestibility, mineral release, and potential glycaemic moderation	Malty aroma, softer texture, and improved porridge smoothness	Prolonged germination may cause grassy flavour, browning, nutrient loss, or microbial contamination	[4,22]
Extrusion	Feed moisture, barrel temperature, screw speed, feed rate, and residence time	Disrupts starch–protein networks; reduces some antinutrients; may release bound phenolics but degrade heat-sensitive compounds	Improved product functionality, starch transformation, digestibility modulation, and snack convenience	Crispness, expansion, roasted aroma, and reduced grittiness	Severe extrusion may reduce lysine, darken colour, lower expansion, or produce hard texture	[23,59]
Roasting or cooking	Temperature, time, particle size, and moisture	Reduces trypsin inhibitors and some tannins; may promote Maillard reactions and phenolic polymerisation	Improved safety and digestibility when controlled	Enhanced roasted aroma, colour, and flavour	Overheating may reduce lysine, increase bitterness, or reduce bioactive availability	[38,57]

**Table 8:** Important research gaps and interdisciplinary needs in Cereal–legume functional foods.

Gap	Research Need	Practical Outcome
Processing frameworks	Lack of relationship between parameter and outcome (e.g., fermentation vs extrusion effects on glycaemic response and SCFA production).	Standardised processing guidelines for functional cereal–legume foods.
Matrix characterisation	Insufficient knowledge about the molecular-level interaction between starch, proteins, and polyphenols in food matrices.	Improved design of foods with predictable bioavailability and metabolic functionality.
Clinical validation	Overreliance on in vitro studies that do not consider microbiome variability.	Human clinical trials confirming metabolic and microbiome effects.
Sensory–health integration	Limited studies link sensory optimisation with metabolic functionality.	Consumer-acceptable functional foods with validated health benefits.

## Data Availability

No new data were generated or analysed in this study. All information presented in this study was obtained from previously published sources, which have been appropriately cited.
